# Targeted-Amplicon NGS for *Blastocystis* sp. in Shepherd Dogs of Portugal Discriminates Co-Colonization with Multiple Zoonotic Subtypes

**DOI:** 10.3390/vetsci12040325

**Published:** 2025-04-02

**Authors:** Sara Gomes-Gonçalves, Maria João Feiteiro, Guilherme Moreira, Rita Cruz, Fernando Esteves, Helena Vala, João R. Mesquita

**Affiliations:** 1 School of Medicine and Biomedical Sciences (ICBAS), Universidade do Porto (UP), 4050-313 Porto, Portugalgmoreiravet@gmail.com (G.M.); 2 Instituto Politécnico de Viseu, Escola Superior Agrária de Viseu, Campus Politécnico, 3504-510 Viseu, Portugal; 3 Epidemiology Research Unit (EPIUnit), Instituto de Saúde Pública, Universidade do Porto (UP), 4050-600 Porto, Portugal; 4 CERNAS-IPV Research Centre, Instituto Politécnico de Viseu, Campus Politécnico, Repeses, 3504-510 Viseu, Portugal; 5 Centre for the Research and Technology of Agro-Environmental and Biological Sciences (CITAB), University of Trás-os-Montes e Alto Douro, 5001-801 Vila Real, Portugal; 6 Associate Laboratory for Animal and Veterinary Science (AL4AnimalS), 1300-477 Lisboa, Portugal; 7 Centro de Estudos de Ciência Animal (CECA), Instituto de Ciências, Tecnologias e Agroambiente (ICETA), Universidade do Porto (UP), Rua D. Manuel II, Apartado 55142, 4051-401 Porto, Portugal

**Keywords:** *Blastocystis*, shepherd dog, zoonotic subtypes, ONT

## Abstract

Research on endoparasites in pet dogs has been growing, but shepherd dogs have largely been overlooked. These dogs frequently share close proximity not only with sheep, which are reservoirs of zoonotic subtypes of *Blastocystis*, but also with their owners. This close contact increases the potential for shepherd dogs to act as intermediates in the transmission of *Blastocystis*. To clarify the role of these dogs as reservoirs for this parasite, this study investigated the presence of *Blastocystis* in shepherd dogs. Stool samples from Portuguese shepherd dogs were analyzed using SYBR-Green-based real-time PCR and melting curve analysis followed by targeted-amplicon NGS for mixed infections detection. Our results revealed a 60% occurrence of *Blastocystis* sp. in shepherd dog stools and frequent identification of zoonotic subtypes ST1–ST4 and ST14. Additionally, we observed mixed infections and subtype diversity within individual dogs, suggesting a potential role in cross-species transmission between livestock and humans.

## Simple Summary

Shepherd dogs, often in close contact with both sheep and humans, may play a role in transmitting *Blastocystis*, a parasite with zoonotic potential. This study analyzed stool samples from Portuguese shepherd dogs coupling Sanger sequencing and NGS. The results showed a high *Blastocystis* occurrence (60%), with common zoonotic subtypes (ST1–ST4 and ST14) and mixed infections. These findings suggest that shepherd dogs could act as intermediaries in cross-species transmission between livestock and humans.

## 1. Introduction

*Blastocystis* sp., a stramenopile, is likely the most prevalent parasite found in the human gut, with estimates suggesting one to two billion global infections [1]. Despite its discovery over a century ago, there is still debate regarding its pathogenic role [1,2]. This organism has been identified across a diverse range of hosts, including wild and domesticated animals [3]. Transmission predominantly occurs via the fecal–oral route, with infections arising from the accidental ingestion of infective cysts [4]. Direct contact with infected individuals (human-to-human or animal-to-human and vice versa) or consuming contaminated food or water are common pathways for infection [5].

While disease by *Blastocystis* is often asymptomatic, particularly in healthy individuals, it may cause more severe presentations in immunocompromised, naïve, or vulnerable hosts [6]. Symptoms associated with its presence include non-specific gastrointestinal issues such as diarrhea, abdominal discomfort, bloating, flatulence, constipation, and, occasionally, skin lesions [6,7]. However, determining whether *Blastocystis* is genuinely pathogenic remains a challenge, given its widespread occurrence and frequency in asymptomatic individuals [8].

Molecular analyses have revealed at least 44 distinct subtypes (STs) of *Blastocystis* sp., based on small subunit ribosomal RNA (SSU rRNA) sequencing [9,10]. Of these, 17 subtypes (ST1–ST10, ST12, ST14, ST16, ST23, ST26, ST35, and ST41) have been linked to human infections, with the remainder predominantly found in animal hosts [11]. Interestingly, among the 17 subtypes capable of infecting humans, ST1–ST4 represent 91.65% of cases, likely due to the efficiency of human-to-human transmission [6,12]. *Blastocystis* sp. has also been detected in companion animals, including pet dogs, with subtypes ST1–ST8, ST10, ST23, and ST24 reported [13]. Among these, ST3 is the most common, accounting for 41.3% of cases, followed by ST2 (39.3%), ST1 (30.9%), ST4 (13.4%), ST8 (12.7%), ST10 (11%), and ST5 (8.1%). Notably, ST23 and ST24 have only been reported in a single study involving dogs [13,14], highlighting a distinct and yet enigmatic role of dogs in *Blastocystis* transmission.

Research on endoparasites in companion animals has increased significantly, particularly regarding their role in transmitting zoonotic parasites, including nematodes, cestodes, trematodes, and protists [15]. However, studies have largely overlooked shepherd dogs, which are necessary in agricultural settings. Their close interactions with sheep, known hosts for various zoonotic protist parasites, position them as intermediates in the transmission dynamics of these pathogens [16,17]. In fact, the frequent contact between shepherd dogs and sheep is known to facilitate the potential sharing of parasites, which can impact both animal and human health [18]. Unlike pets that typically have limited exposure to livestock, shepherd dogs work in agricultural settings and often interact with sheep and other farm animals. Sheep are established reservoirs of human-infective *Blastocystis* STs, hosting a range of zoonotic subtypes (ST1–ST5, ST7, ST10, ST12, ST14, ST15, ST21, ST23, ST24, and ST26) [16]. Regular contact with sheep feces and contaminated grazing areas may place shepherd dogs at an elevated risk of infection, particularly with zoonotic subtypes.

Despite the growing recognition of *Blastocystis* in various animal hosts, there is a significant gap in understanding its occurrence specifically in shepherd dogs. Unlike companion dogs, which typically have controlled environments and limited exposure to livestock, shepherd dogs live and work in agricultural settings, where they experience constant and direct contact with sheep. Given that sheep are known reservoirs of zoonotic *Blastocystis* subtypes (ST1–ST5, ST7, ST10, ST12, ST14, ST15, ST21, ST23, ST24, and ST26), shepherd dogs may play a role in the epidemiology of this parasite. This makes them a distinct and important target population for study as they may act as intermediary hosts between livestock and humans, a role not commonly associated with pet dogs. Additionally, the close contact that shepherd dogs have with both sheep and humans, combined with previous detections of *Blastocystis* in Portuguese patients [19,20], underscores the need for further investigation in order to shed light on the transmission routes of *Blastocystis* sp. to humans.

This study aims to fill a gap in international literature by providing the first molecular identification and characterization of *Blastocystis* sp. in shepherd dogs. While *Blastocystis* has been detected in companion dogs in previous studies [13,21,22,23], limited data exist on working shepherd dogs that interact closely with livestock. Our study differentiates itself from previous works by employing a combined methodological approach coupling SYBR-Green-based real-time PCR followed by targeted amplicon sequencing with Oxford Nanopore Technologies (ONT) to enhance subtype detection and evaluate the possibility of mixed infections. This approach allows for greater resolution in identifying *Blastocystis* subtypes compared to conventional techniques. Furthermore, by analyzing shepherd dogs from an agricultural context, our research contributes new insights into the potential zoonotic role of working shepherd dogs in *Blastocystis* transmission.

## 2. Materials and Methods

### 2.1. Sampling

**Figure 1:**
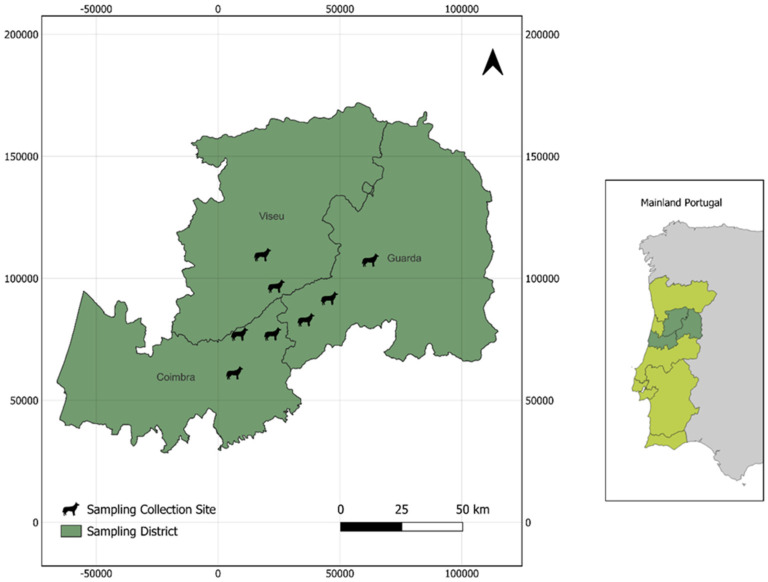
The map illustrates the spatial distribution of sampling collection sites (indicated by black dog icons) across the districts of Coimbra, Viseu, and Guarda in Central Portugal. Portugal mainland is represented in light green while districts where samples were collected are shaded in dark green. An inset map of Mainland Portugal (right) highlights the location of the sampling districts within the national context. The scale bar, displayed in kilometers, provides a reference for distance, while the north arrow in the upper-right corner indicates map orientation.

A total of 50 stool samples were collected after defecation and stored at −20 °C. The samples were collected from different breeds of shepherd dogs, including Border Collie, Serra de Aire Dog, Transmontano Cattle Dog, Serra da Estrela Dog, and mixed breeds, which were classified as mix or not defined (ND). The samples were gathered from the central region of Portugal, specifically from Arganil, Celorico da Beira, Gouveia, Nelas, Oliveira do Hospital, Tábua, and Viseu. A detailed description of the sampling is provided in Table 1, while Figure 1 illustrates the geographical distribution of the samples.

### 2.2. DNA Extraction

DNA extraction was performed by diluting stool samples to 10% in phosphate-buffered saline (PBS) at a pH of 7.2, with 500 μL of PBS added to each fecal swab. The mixtures were vortexed thoroughly and centrifuged at 8000× *g* for 5 m (Eppendorf, Hamburg, Germany). Following centrifugation, 140 μL of the supernatant was used for DNA extraction and purification using the QIAamp DNA Mini Kit (Qiagen, Hilden, Germany) in accordance with the manufacturer’s instructions. Automated extraction was conducted using the QIAcube^®^ platform (Qiagen). The extracted DNA was stored at −80 °C in RNase-free water for later analysis.

### 2.3. Molecular Detection of Blastocystis sp.

Molecular detection of *Blastocystis* sp. was carried out using real-time SYBR Green PCR (qPCR) as described by [24]. The primer pair BL18SPPF1/BL18SR2PP (STABVIDA, Costa da Caprica, Portugal), targeting the 18S small subunit ribosomal RNA (SSU rRNA) gene, was used to amplify a fragment of approximately 300 bp. PCR reactions were performed on a T100 thermocycler (Bio-Rad, Hercules, CA, USA). Reaction mixtures were prepared using the Xpert Fast SYBR (Uni) Blue mix (GRiSP^®^, Porto, Portugal), following the manufacturer’s protocol.

### 2.4. Sanger and Oxford Nanopore Technologies (ONT) Sequencing

Amplicons showing the expected melting temperatures were purified using the GRS PCR and Gel Band Purification Kit (GRiSP^®^, Porto, Portugal) and were bilateral sequenced via Sanger sequencing. The resulting sequences were edited, aligned, and analyzed using the BioEdit Sequence Alignment Editor (v7.2.5). The consensus sequences were compared to the NCBI GenBank database using BLASTn (https://blast.ncbi.nlm.nih.gov/Blast.cgi, accessed on 30 March 2025). In cases where Sanger sequencing produced unreadable results, amplicons were sequenced with Oxford Nanopore Technologies. This was carried out using the PromethION 24 platform (ONT, Oxford, UK (Oxford Nanopore Technologies)) running software version 19.06.9 (MinKNOW GUI v4.0.23). The R10.4.1 flow cell was used to enhance read accuracy through advanced pore design and chemistry. Library preparation was performed with the Native Barcoding Kit 96 V14 (SQK-NBD114.96).

### 2.5. Control Procedures

To ensure the reliability and validity of the results, control measures were implemented throughout the study, covering all stages from sample collection to molecular analysis and sequencing. Regarding the stool, samples were collected from shepherd dogs using sterile equipment. Only the top portion of the stool was collected to avoid soil contamination, and samples were immediately stored at −20 °C to prevent DNA degradation.

For DNA extraction, a negative control, consisting of molecular grade RNAase-free water, was included to detect any potential contamination during the extraction process. A positive control, consisting of a known *Blastocystis* sp. ST10 sample from a previous study [25], was also processed alongside the stool samples to verify the effectiveness of the extraction procedure. DNA was extracted using the QIAamp DNA Mini Kit (Qiagen, Hilden, Germany) with the automated QiaCube^®^ platform (Qiagen, Hilden, Germany), and the extracted DNA was stored at −80 °C for future analysis.

During the qPCR amplification using Xpert Fast SYBR (Uni) Blue mix (GRISP^®^, Porto, Portugal), several controls were used to ensure the accuracy of the results. A No-Template Control (NTC), consisting of all PCR reagents without DNA, was included to detect any potential contamination during amplification. A positive PCR control containing known *Blastocystis* sp. DNA was also included. qPCR reactions were conducted on T100 thermocycler (Bio-Rad, Hercules, CA, USA).

For sequencing, Sanger sequencing was performed on amplicons purified using the GRS PCR and Gel Band Purification Kit (GRiSP^®^), with positive controls known to contain *Blastocystis* sp. included to verify the accuracy of the sequencing process. In cases where Sanger sequencing yielded ambiguous results, Oxford Nanopore Technologies (ONT) was used with the PromethION 24 platform (ONT, Oxford, UK). Barcoded sequencing kits were employed to minimize the risk of cross-contamination, and library preparation was conducted using the Native Barcoding Kit 96 V14 (SQK-NBD114.96).

### 2.6. Bioinformatic Analysis

The raw FASTQ reads were basecalled using ont-doradod-for-promethion v7.4.12 in super-accurate mode, with a minimum Q-score of 10. Adapter and barcode trimming were performed using MinKNOW, and sequences shorter than 200 bp or longer than 350 bp were filtered out with NanoFilt v2.8.0 [26]. Clustering of reads was conducted using the vsearch -cluster_fast command, applying a 98% identity threshold. Consensus sequences were polished using Racon v1.5.0 [27], with alignment files generated by Minimap2 v2.28-r1209 [28]. Subtyping was carried out using PARSID v1.0.0 [29], referencing the curated *Blastocystis* sp. database (http://entamoeba.lshtm.ac.uk/ref.blasto.txt, accessed on 15 November 2024). A cut-off BLAST score of 98% and sequence divergence thresholds of 98% were applied. Results with query coverage exceeding 98% and identity greater than 98% were included [24,30]. The Sanger sequences and the highest-ranking reads for each subtype were deposited in GenBank under accession numbers PQ591423–PQ591460, and the raw FASTQ files were submitted to the NCBI Sequence Read Archive under project number PRJNA1184492.

### 2.7. Phylogenetic Analysis

Phylogenetic analysis was performed by including 43 full-length sequences retrieved from the *Blastocystis* sp. curated database (http://entamoeba.lshtm.ac.uk/ref.blasto.txt, accessed on 15 November 2024). Sequence alignment was carried out using MAFFT v7.490, employing the L-INS-i method for accuracy with diverse sequences. A Maximum Likelihood tree was constructed with IQ-TREE, using 1000 bootstrap replicates and the K2P + I + G4 substitution model, in line with reference studies. The phylogenetic tree was rooted on the ST15/ST28 cluster, which represents an early-diverging lineage of *Blastocystis* [12,31,32,33]. Additional annotations and visual enhancements were created using the Interactive Tree of Life (iTOL) platform v7.

### 2.8. Statistical Analysis

The occurrence of *Blastocystis* sp. in shepherd dogs was calculated as the proportion of positive samples relative to the total number analyzed, with a 95% confidence interval (95% CI). Data processing and preliminary analyses were performed using Microsoft Excel^®^ for Microsoft 365 MSO (Version 2312, Build 16.0.17126.20132, 64-bit).

## 3. Results

**Figure 2:**
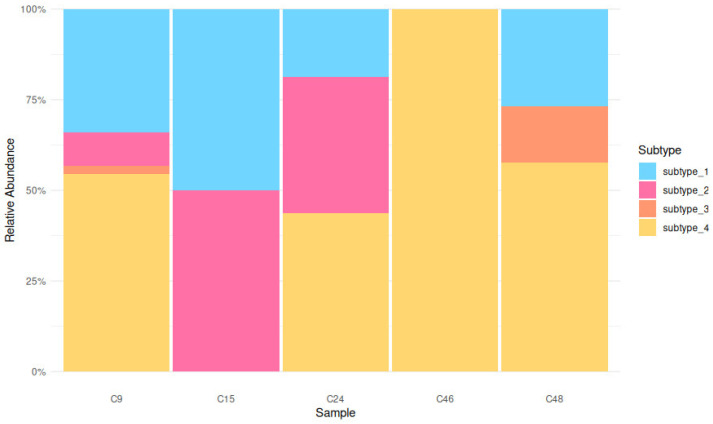
Relative abundance of *Blastocystis* subtypes in shepherd dog stool samples. The bar plot represents the distribution of four subtypes (subtype 1–4) across five samples (C9, C15, C24, C46, and C48). Colors indicate different subtypes: subtype 1 (blue), subtype 2 (pink), subtype 3 (orange), and subtype 4 (yellow). The y-axis represents the relative abundance of each subtype within a sample, highlighting mixed infections and variation in subtype composition.

**Figure 3:**
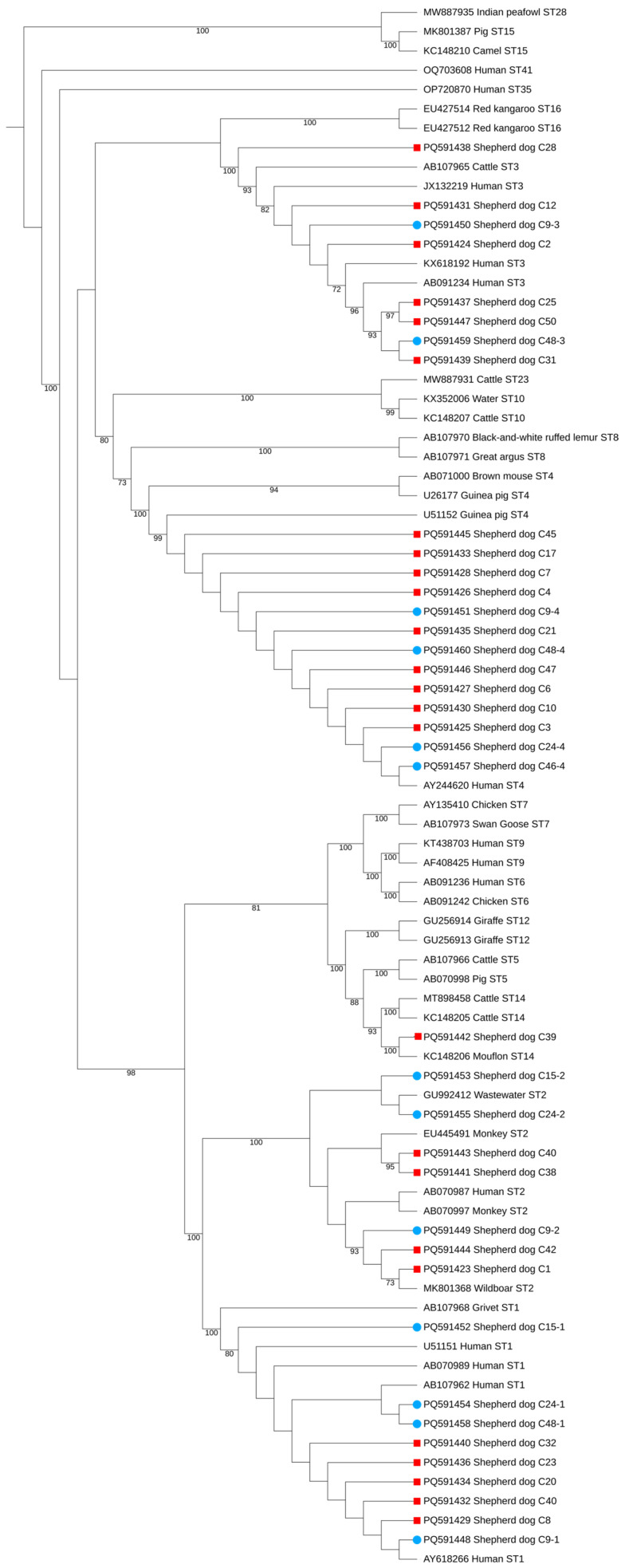
Phylogenetic tree of *Blastocystis* sp. subtypes (STs) was constructed using full-length SSU rRNA gene sequences. The sequences generated in this study are represented with distinct markers. Squares denote sequences obtained via Sanger sequencing, while circles indicate those derived from Oxford Nanopore Technologies (ONT). The tree was obtained using IQ-TREE, employing the K2P + I + G4 substitution model with 1000 bootstrap replicates to ensure robust inference. Only bootstrap values of 70% or higher are shown in the figure.

In this study, of the 50 shepherd dog stool samples, 30 were positive for at least one *Blastocystis* sp. ST, yielding an occurrence of 60% (30/50; 95% confidence interval [CI]: 45.18–73.59). Of those, 25 showed single-peaked chromatograms by Sanger sequencing, and BLASTn analysis of these allowed the classification of *Blastocystis* ST1–ST4. Five, four, six, and ten dogs were positive for *Blastocystis* ST1–ST4, respectively. The remaining five presumptively showed mixed infections by visual inspection of chromatograms. These were further analyzed by targeting the 18S small subunit ribosomal RNA using the ONT platform, which confirmed mixed infections in four of the five cases. The relative abundance of subtypes in these mixed infections is shown in Figure 2. Further details on the geographical distribution, subtypes, and herd sizes are provided in Table 2. All the subtypes found in this study were all zoonotic subtypes with the ST4 being the most found (*n* = 13), followed by ST1 (*n* = 9), ST2 (*n* = 8), ST3 (*n* = 8), and ST14 (*n* = 1). Figure 3 presents the phylogenetic tree confirming the identified subtypes. For samples with mixed infections, a representative sequence was selected for each subtype within each sample.

## 4. Discussion

*Blastocystis* sp. is one of the most common intestinal parasites in humans, with an estimated global prevalence of one to two billion infections. However, its pathogenic role remains unclear as it is often found in asymptomatic individuals. Regional differences in infection prevalence across Europe have been noted, including 3.4% in France [34], 1.8% in Czech Republic [35], 1.5% in Turkey [36], and 21% in Italy [37]. Notably Greece [38] and Spain [21] did not report positive cases, scoring occurrences notably lower than the 60% found in the present study. These variations suggest that environmental conditions, husbandry practices, and host diversity may influence the spread of *Blastocystis*. Despite this, the role of domestic dogs as potential reservoirs has not been fully explored. This study aims to address this gap by investigating *Blastocystis* infections in shepherd dogs in Portugal, marking the first report of the parasite in this population.

Our study found that 60% (30/50) of the shepherd dogs sampled were infected, and all positive samples contained zoonotic subtypes, particularly ST1–ST4, which account for approximately 90% of global *Blastocystis* infections in humans. These findings suggest that shepherd dogs, due to their regular contact with both livestock and humans, may act as a link for cross-species transmission. The presence of multiple zoonotic subtypes within some dogs, as revealed by ONT sequencing, further supports this hypothesis and highlights the potential for transmission between dogs, sheep, and humans. This is consistent with the existing literature, which suggests that domestic animals, such as dogs and sheep, can harbor a plethora of *Blastocystis* subtypes, increasing the risk of cross-species transmission [3,13,39]. Additionally, studies have shown a higher prevalence of *Blastocystis* in dog owners compared to non-owners (25% vs. 11.5%) [3], reinforcing the role of domestic dogs as potential *Blastocystis* reservoirs.

However, there are some limitations in this study. The small sample size in certain regions (e.g., Gouveia, Nelas, and Tábua) and among specific dog breeds (e.g., Transmontano Cattle Dog and mixed breeds) limits the ability to draw definitive conclusions about geographic or breed-specific infection patterns. Additionally, the absence of samples from sheep and humans in close contact with shepherd dogs limits the ability to fully determine their role in cross-species transmission, which could provide clearer evidence of dogs as intermediaries. While mixed infections were observed, further research is required to determine if they are more common in specific breeds or linked to environmental factors. Larger sample sizes from a wider range of regions and dog breeds will help clarify these aspects.

The presence of zoonotic *Blastocystis* subtypes in shepherd dogs raises concerns about their potential role in the transmission of *Blastocystis* between sheep, humans, and other animals. Given their proximity to both livestock and humans, shepherd dogs may facilitate the spread of zoonotic strains, especially in rural and agricultural areas where human–animal interactions are frequent. Monitoring *Blastocystis* infections in shepherd dogs could provide valuable insights into transmission pathways and help manage associated public health risks.

Although a 2024 study confirmed that dogs are not natural hosts of *Blastocystis* typically found in herbivores and omnivores [40]. Our findings refute those by suggesting that shepherd dogs are susceptible to a wide range of *Blastocystis* subtypes, including zoonotic strains, and may act as bridges for cross-species transmission from sheep to humans and other species. While the pathogenicity of *Blastocystis* is still debated, its association with gastrointestinal symptoms and other health issues, such as urticaria, in humans suggests that it should not be dismissed as harmless. The presence of zoonotic strains in shepherd dogs underscores the importance of considering domestic animals as potential reservoirs.

## 5. Conclusions

This study provides the first report of *Blastocystis* in shepherd dogs in Portugal, identifying a notable prevalence (60%) of zoonotic subtypes. While these findings suggest that shepherd dogs, due to their frequent exposure to livestock, particularly sheep, may facilitate cross-species transmission of *Blastocystis*, further research is required to confirm their potential role as reservoirs and assess the actual risk to human health. Future studies should focus on comparing *Blastocystis* subtypes found in shepherd dogs, sheep, and humans who have close contact with both to clarify transmission dynamics and better understand the public health implications of these findings.

## Figures and Tables

**Table 1:** Distribution of sampled animal breeds across municipalities. Each municipality is listed alongside the specific breeds sampled within that area, with the number of individuals for each breed noted in parentheses.

Municipality	Breed (No. of Individuals)	No. of Samples (*n*)
Arganil	SE (1)	1
Celorico da Beira	BC (1), SE (1), ND (1)	3
Gouveia	BC (1), SE (18), ND (2)	21
Nelas	BC (2), BC × SA (Mix) (1), SA (2), SE (7)	12
Oliveira do Hospital	GT (1), SA (3), SE (4)	8
Seia	SE (2), ND (1)	3
Tábua	SE × ND (Mix) (1)	1
Viseu	SE (1)	1
Total	BC (4), GT(1) SA (5), SE (34), ND (4), Mix (2)	50

BC—Border Collie, GT—Gado Transmontano, SA—Serra de Aire, SE—Serra da Estrela, ND—not defined, Mix—mixed breeds.

**Table 2:** *Blastocystis* subtypes identified in shepherd dogs across different municipalities in Central Portugal. The table presents the distribution of *Blastocystis* subtypes (ST) detected in individual shepherd dog samples, along with the corresponding municipality and herd size.

Municipality	Sample ID	Subtype (ST)	Size of Herd (*n*)
Arganil	C20	ST1	79
Celorico da Beira	C31	ST3	250
Gouveia	C48	ST1, ST3, ST4	190
	C32	ST1	134
	C40	ST2	713
	C4	ST4	111
	C24	ST1-ST3	49
	C15	ST1-ST2	47
	C13	ST1	19
	C50	ST3	190
	C38	ST2	197
	C48	ST14	99
	C28	ST3	99
	C1	ST2	31
	C2	ST3	31
Nelas	C9	ST1-ST4	700
	C7	ST4	700
	C8	ST1	700
	C6	ST4	700
	C47	ST4	200
	C46	ST4	200
	C10	ST4	700
	C45	ST4	91
Oliveira Hospital	C23	ST1	11
	C21	ST4	11
	C42	ST2	15
	C25	ST3	90
Seia	C17	ST4	90
Tábua	C3	ST4	3
Viseu	C12	ST3	10

## Data Availability

The data presented in this study are available on request from the corresponding author.
